# Graphene-Like Porous ZnO/Graphene Oxide Nanosheets for High-Performance Acetone Vapor Detection

**DOI:** 10.3390/molecules24030522

**Published:** 2019-01-31

**Authors:** Hongwu Wang, Ding Wang, Liang Tian, Huijun Li, Ping Wang, Nanquan Ou, Xianying Wang, Junhe Yang

**Affiliations:** 1 School of Materials Science and Engineering, University of Shanghai for Science & Technology, No. 516 JunGong Road, Shanghai 200093, China; 162522318@st.usst.edu.cn (H.W.); wangding@usst.edu.cn (D.W.); tl502004571@126.com (L.T.); huijunli0701@126.com (H.L.); pingwang2050@outlook.com (P.W.); ounanquan@163.com (N.O.); 2 Shanghai Innovation Institute for Materials, Shanghai 200444, China

**Keywords:** ZnO/GO nanosheets, nanocomposites, acetone gas sensors, two-dimensional structure

## Abstract

In order to obtain acetone sensor with excellent sensitivity, selectivity, and rapid response/recovery speed, graphene-like ZnO/graphene oxide (GO) nanosheets were synthesized using the wet-chemical method with an additional calcining treatment. The GO was utilized as both the template to form the two-dimensional (2-D) nanosheets and the sensitizer to enhance the sensing properties. Sensing performances of ZnO/GO nanocomposites were studied with acetone as a target gas. The response value could reach 94 to 100 ppm acetone vapor and the recovery time could reach 4 s. The excellent sensing properties were ascribed to the synergistic effects between ZnO nanosheets and GO, which included a unique 2-D structure, large specific surface area, suitable particle size, and abundant in-plane mesopores, which contributed to the advance of novel acetone vapor sensors and could provide some references to the synthesis of 2-D graphene-like metals oxide nanosheets.

## 1. Introduction

With the aggravation of air pollution, volatile organic compounds (VOCs) become one of the most intractable issues. Acetone, as a widely used organic solvent, is hazardous and can cause fatigue, narcosis, and eye irritation [1,2]. In the chemical industry, the concentration of acetone in the workplace is limited to less than 450 mg/m^3^ within a short time by national regulation. However, acetone is so volatile that the concentration can exceed this regulation easily while humans cannot perceive that. Therefore, the superior efficiency acetone vapor sensor plays an important role in the workplace for personal safety [3,4]. The detection of trace acetone can predict and prevent related diseases in advance, which has profound significance in human health maintenance. Hence, it is of great significance to monitor the surrounding acetone concentration in the area of chemical industry, environment, and security.

Acetone sensors based upon metal oxide semiconductors (MOS), such as ZnO, WO_3_, and SnO_2_ have been widely studied due to their excellent sensitivity, unique selectivity, low cost, and simplicity of the manufacturing process [5,6]. In general, the response of n-type MOS is based upon the change of the electron depleted layers’ width at various gas atmospheres [7]. As a representative n-type semiconductor, ZnO is environment friendly and usually has a high response value to oxidizing or reducing gases. Various ZnO nanostructures have been successfully synthesized such as nanowires, nanosprings, nanorings, and nanocombs [8]. Compared with other nanostructures, graphene-like porous ZnO nanosheets are of a large surface area, smaller particle size, and controlled crystallographic plane, which is helpful in improving the sensing performance [9].

The sensitive property of MOS can be further modified by combining with graphene materials, for instance, carbon nanotubes, graphene, graphene oxide (GO), and reduced graphene oxide (rGO) [10,11,12]. As a representative of single layer carbon-based materials, GO is of a unique 2-D structure, remarkable electronic properties, simple fabrication, large surface area, and abundant surface functional groups [13,14]. Many preparation methods such as the hydrothermal method, ionic layer adsorption and reaction method, and physical mixing method are used to prepare MOS/GO composites [15,16,17]. However, the loose adhesion between MOS and GO is adverse to electron transport. Through controlling the thermal treatment conditions, in-situ modification of MOS with GO can be realized. The reason why the new MOS/GO nanocomposites are expected to be widely used is that the large specific surface area, unique electrical properties, and abundant surface adsorption sites.

In this work, 2-D ZnO/GO nanocomposites were synthesized via a facile wet-chemical method, utilizing GO as both the template and sensitizer. The morphology and microstructure composition of these samples were studied through various material characterization methods. The sensitive properties of a series of ZnO/GO nanosheets were comparatively investigated through a gas sensor intelligent test meter. The sensitivity, selectivity, and response/recovery times were studied by the gas sensitivity test curves. The proportion of GO in composites was controlled by changing the calcining temperatures, and their effects on the sensing performance were comprehensively studied.

## 2. Results and Discussions

### 2.1. Materials Characterization

**Figure 1:**
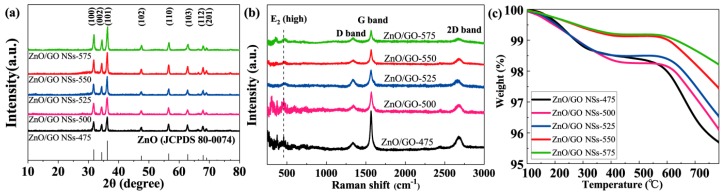
(**a**) XRD patterns of ZnO/GO NSs-T; (**b**) Raman spectra of ZnO/GO NSs-T; (**c**) TGA curves of ZnO/GO NSs-T.

Figure 1a shows the XRD patterns of ZnO/GO NSs-T synthesized under different calcining temperatures. All the diffraction peaks (100), (002), (101), (102), and (110) of XRD patterns revealed the hexagonal wurtzite phase of ZnO [18]. Diffraction peaks of GO were not found due to its small amount. It is noteworthy that the shape and strength of diffraction peaks were similar for different samples, and indicated the high thermal stability of ZnO in nanocomposites. The crystallite size of the samples was estimated by the Scherrer Equation (1) [19].

(1) $$D=\frac{K\;\gamma }{\beta \;cos\theta }$$

where D is the crystallite size, *β* is the full width at half maximum, *θ* is the diffraction angle, and *λ* is the wavelength of X-ray. The crystallite sizes of the five samples were estimated to be 16 ± 2 nm after calcining at different temperatures. Generally, the resistance variation of metal oxide nanostructure under reducing gases can reach a maximum if its grain size is lesser or equal to the Debye length (λ_D_) distance, because in this case, nanosheets could deplete the electrons in the air completely [20]. As reported, the λ_D_ of ZnO is 20–30 nm, which indicated that the ZnO/GO NSs-T might have excellent gas sensitivity to reducing gases [21].

The structure and the chemical state of GO were researched by Raman spectroscopy [22]. The ZnO/GO NSs-T after annealing was characterized by Raman spectrometer excited with a 532 nm laser line. As Figure 1b shows, all spectra showed three main strong peaks at 1336 cm^−1^, 1568 cm^−1^, and 2681 cm^−1^, they are D band, G band, and 2-D band of GO, respectively, which clearly proved the existence of GO in the nanocomposites [23]. The D band is related to the chaotic structure of GO, such as the graphene ends, the defects in GO sheets, and finite size crystalline domains of the graphene together with the contribution of impurities (such as amorphous carbon) in GO. In contrast, the G band stands for the sp^2^ hybridized carbon atom structure. The ratios between the intensity of D and G band (I_D_/I_G_) were calculated to measure the changing of GO by heat treatment. The I_D_/I_G_ ratios of ZnO/GO NSs-475, ZnO/GO NSs-500, ZnO/GO NSs-525, ZnO/GO NSs-550, and ZnO/GO NSs-575 were 0.155, 0.214, 0.353, 0.363, and 0.391, respectively. As the annealing temperature increases, the value of I_D_/I_G_ also increases because of the defects or cavity sites formed on the GO [24]. Furthermore, the Raman peaks of ZnO were also observed and corresponded to the E_2_ high mode of the wurtzite structure vibrations [25]. The amount of GO in the ZnO/GO NSs-T was estimated by TGA curves. Before testing, the samples were kept heated at 100 °C for 20 min to ensure the solvent evaporated and all the weight loss of samples were attributed to the decrement of GO. As Figure 1c shows, the weight loss of ZnO/GO NSs-T decreased from 4.3% to 1.7%, while the heating temperature changed from 475 °C to 575 °C, which indicated the regulation ability of the GO amount in the ZnO/GO nanocomposites.

**Figure 2:**
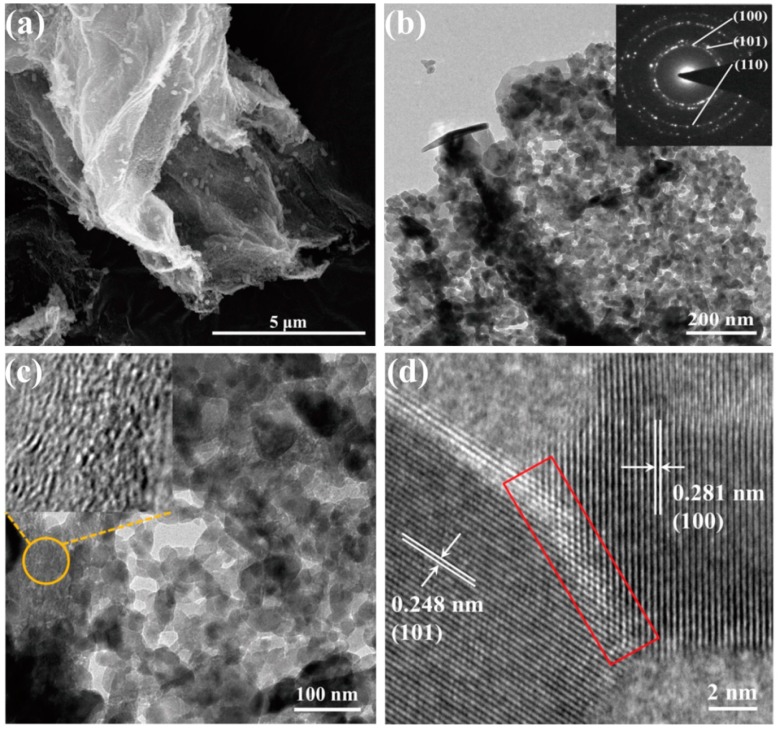
(**a**) The SEM image of ZnO/GO NSs-525; (**b**) TEM image of ZnO/GO NSs-525 and the selected area electron diffraction (SAED) pattern was inset; (**c**) large magnification TEM image of ZnO/GO NSs-525, the inset is an enlarged disordered lattice fringe view of GO in the local area of a HRTEM image; (**d**) HRTEM image of ZnO/GO NSs-525.

The SEM image of ZnO/GO NSs-525 is shown in Figure 2a. The morphology of ZnO/GO NSs-525 was graphene-like mesoporous nanosheets that were made up of the arranged nanoparticles. In order to clearly observe the constituent particle size and mesopores of the ZnO/GO NSs-525, a TEM test was performed. As shown in Figure 2b, the 2-D nanosheets consisted of in-plane mesopores with interconnected nanoparticles. The inset of Figure 2b is a selected area electron diffraction (SAED) pattern which indicates a polycrystalline structure. The pore diameter and particle size could be obtained from Figure 2c, and the values in the ranges of 15–23 nm and 20–40 nm, respectively. The existence of GO was further proved by the disordered lattice fringes in the inset image of Figure 2c. HRTEM images in Figure 2d showed distinct lattice fringes, and the lattice spacing was 0.281 nm and 0.248 nm, respectively, corresponding to the (100) and (101) planes of ZnO [26].

**Figure 3:**
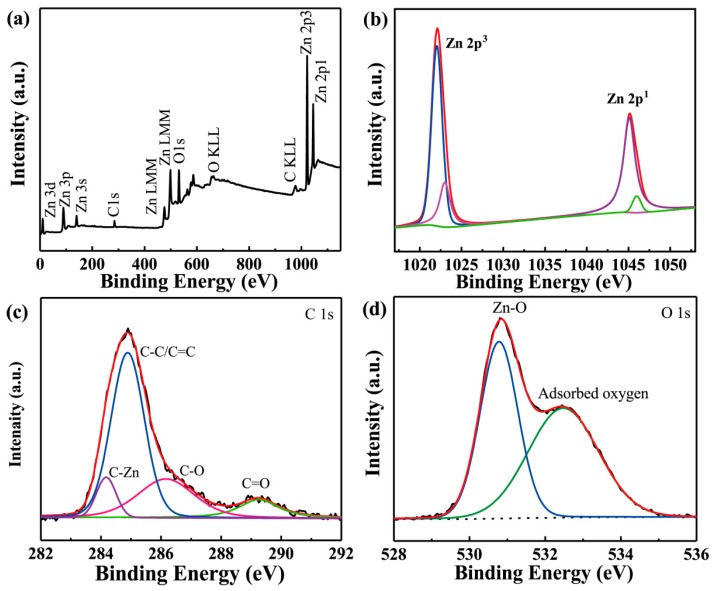
(**a**) XPS survey spectrum of the ZnO/GO NSs-525; high-resolution core-level XPS spectra: (**b**) Zn 2p, **(c)** C 1s and (**d**) O 1s.

The chemical states and surface compositions in sensing materials were studied by XPS. The full survey spectrum of ZnO/GO NSs-525 in Figure 3a indicated the presence of C, O, and Zn elements. There are two peaks, the one corresponding to Zn 2p^3^ is at a binding energy of 1022.1 eV and another at 1045.1 eV is Zn 2p^1^. Two small peaks were shaped by the influence of C-Zn (Figure 3b) [27]. Figure 3c shows four peaks of C1s at 284.1 eV, 284.7 eV, 286.1 eV, 289.1 eV, consistent with the C-Zn, C-O, C-C/C=C, and C=O bands, respectively, which indicates that the intensity of peaks C-O and C=O are strong in GO. The peak of C-Zn determines that the bond C-Zn has successfully formed after annealing treatment [28]. As shown in Figure 3d, an asymmetric O 1s spectrum appears at 530.7 eV, which can be attributed to oxygen ions (O^2−^) in the Zn-O bond of the wurtzite structure [29]. The peak at a binding energy of 532.4 eV was wide and had good symmetry, which was attributed to the oxygen chemisorbed on the surface of ZnO/GO NSs, which is closely related to the gas sensitive properties.

**Figure 4:**
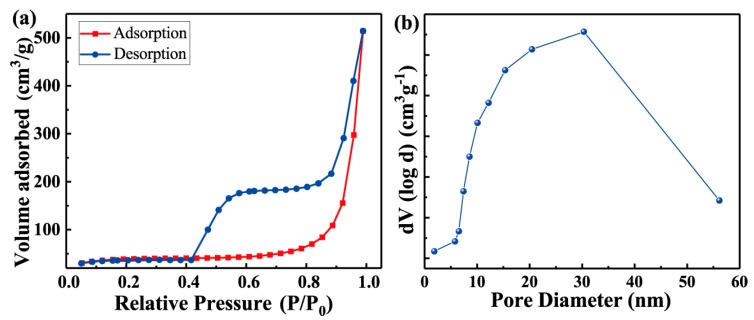
(**a**) Isothermal adsorption/desorption curve, (**b**) pore diameter distribution of ZnO/GO NSs-525.

The porous nature of ZnO/GO nanosheets was further investigated by a nitrogen adsorption survey and the results are shown in Figure 4. The isotherm demonstrated a typical II hysteresis loop while the relative pressure is between 0.42 and 0.95, implying the existence of mesopores [30]. The surface area of the ZnO/GO NSs-525 was determined to be 125.2 m^2^∙g^−1^ by Brunauer–Emmett–Teller calculations. According to the Barrett–Joyner–Halenda (BJH) pore size distribution analysis determined from the desorption branch, the average BJH pore diameter is ~7.7 nm. The pore size distributes in the range of 2~55 nm, along with the in-plane pores observed from the TEM image (Figure 4b and Figure 1b).

### 2.2. Sensing Properties

**Figure 5:**
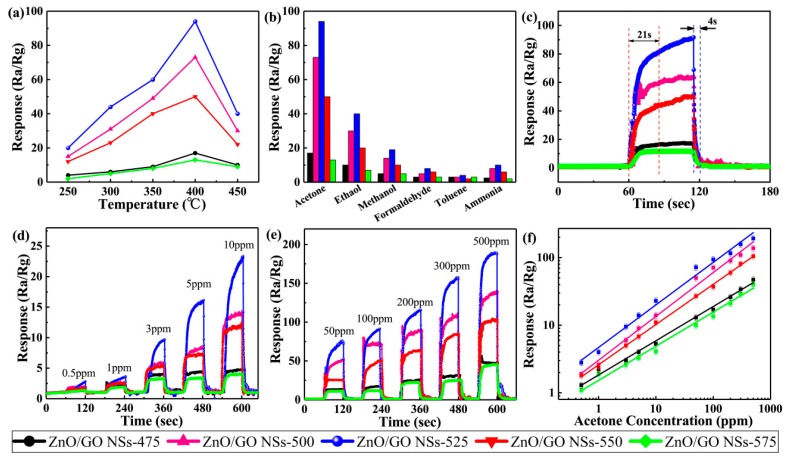
(**a**) Sensor response values of ZnO/GO NSs-T at 250–450 °C towards 100 ppm acetone; (**b**) Responses of ZnO/GO NSs-T sensor to 100 ppm different gases at 400 °C; (**c**) Response/recovery graphs of ZnO/GO NSs-T sensor towards 100 ppm acetone at 400 °C; (**d**) Response-recovery curves of the sensors to different acetone vapor concentrations (0.5–10 ppm); (**e**) Response-recovery curves of the sensors to different acetone vapor concentrations (50–500 ppm); (**f**) the relationship of response value and acetone concentration of ZnO/GO NSs-T sensors.

The operation temperature of sensors has an important effect on gas sensitivity from the aspects of thermodynamics and kinetics [31]. At various temperatures, the gas sensors fabricated by ZnO/GO NSs-T were tested in 100 ppm acetone vapor to identify the optimal operating temperature. As Figure 5a shows, all sensors’ optimum working temperatures to acetone vapor were 400 °C. In addition, the variation tendency of sensing performance was increasing initially and then decreasing with the calcination temperature, and the optimum calcination temperature was found to be 525 °C. It is remarkable that the response value of the ZnO/GO NSs-525 sensor is 94, which was about 5 times larger than that of ZnO/GO NSs-475 and ZnO/GO NSs-575 at the same operating temperature, indicating that a suitable calcinating temperature is the key to a high-efficiency acetone sensor.

In gas detection applications, selectivity is another important factor. The gas sensing response of the ZnO/GO NSs-T to various gases at the same testing condition was shown in Figure 5b. Clearly, the sensitivity to acetone vapor was much better than the response value of the other five kinds of gases: Feckly was 2 times higher than that of ethanol, 5 times that of methanol, and 10 times that of toluene, formaldehyde, and ammonia. Interestingly, the selectivity of ZnO/GO NSs-525 to the interference gases after calcinating at different temperatures is almost unchanged, which indicated that the improvement of gas sensitivity might be due to the material’s structure [32].

Moreover, the response and recovery times are another important parameter in practical application. It was tested by injecting 100 ppm acetone vapor into the cabinet with the ZnO/GO NSs-525 sensors at the optimal temperature (400 °C). The result was shown in Figure 5c. All sensors demonstrated rapid response and recovery properties: The response times are in the ranges of 11–21 s and the recovery times are about 4 ± 1 s, respectively. They are short enough for real-time acetone vapor detection.

Figure 5d,e have shown the response-recovery curves of ZnO/GO NSs-525 sensors to different acetone vapor concentrations (0.5–10 ppm) and (50–500 ppm) at the optimal temperature (400 °C). The response values increased with acetone concentration in the entire range (0.5–500 ppm). Furthermore, to study the relationship between response values and acetone concentrations, the correlation lines between responses of the ZnO/GO nanosheets gas sensors and concentrations of acetone were fitted in Figure 5f. The response and concentration (from 0.5 ppm to 500 ppm) showed a good linear performance of a double logarithmic linear relationship, which is of great importance in practical applications. The relationship of slopes is as follows: ZnO/GO NSs-525 (0.619) > ZnO/GO NSs-500 (0.597) > ZnO/GO NSs-550 (0.586) > ZnO/GO NSs-475 (0.469) > ZnO/GO NSs-575 (0.480). Normally, a metal oxide semiconductor sensor’s response can be empirically calculated from the following Equations (2) and (3) [33]:

S = 1 + αC_g_^β^ (2)

lg(S − 1) = lgα + βlgC_g_ (3)

where α is prefactor, C_g_ is the target gas concentration and β is the surface species charge parameter. According to the reports, the exponent β is 0.5 when the adsorbed oxygen species is O^2−^ ions, and it will be changed to 1 for O^−^ ions. Thus, the adsorbed surface oxygen species of ZnO/GO NSs-525 composite can be evaluated from the β value. The β values for ZnO/GO NSs-525 could be calculated about 0.5 from the response-concentration curves in Figure 5f, indicating that O^2−^ ions were the major absorbed oxygen species on these samples’ surface.

**Figure 6:**
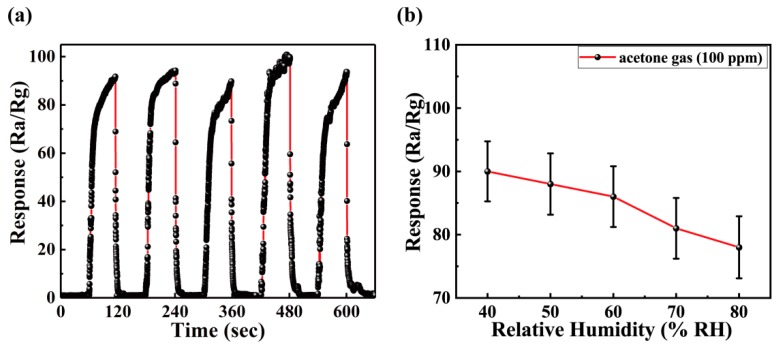
(**a**) Reproducibility of the ZnO/GO NSs-525 to 100 ppm of acetone at 400 °C; (**b**) response values to 100 ppm acetone at different relative humidity.

Furthermore, repeatability and reversibility are considerable parameters for application in practice. The sensing test to 100 ppm acetone was repeated to examine the reversibility and repeatability of the sensor at 400 °C. As shown in Figure 6a, we have tested five periods and the response value of the ZnO/GO NSs-525 sensor only exhibits a tiny change compared with the former test of 100 ppm acetone vapor under optimal temperature; meanwhile, the fast response/recovery speed was still maintained. Of course, the influence of ambient humidity cannot be neglected because the high humidity level may affect the acetone vapor detection signal. Figure 6b shows the response values of the ZnO/GO NSs-525 sensor to 100 ppm acetone vapor at the range of 40–80 %RH. The sensitivity decreased with the relative humidity. However, the response value can still reach 78 at the humidity of 80 %RH, indicating that the material has good anti-humidity interference.

The comprehensive sensing properties, for example, the sensitivity, selectivity, and response/recovery speed are important for the application. In Table 1, the sensing properties of ZnO/GO NSs-525 to acetone vapor detection are compared with Refs [17,34,35,36,37,38,39,40]. Compared with other ZnO-based gas sensors, the ZnO/GO NSs-525 exhibited excellent sensitivity, faster recovery speed, and good selectivity for acetone vapor detection in the 0.5 ppm to 500 ppm.

### 2.3. Sensitive Mechanism

**Scheme 1:**
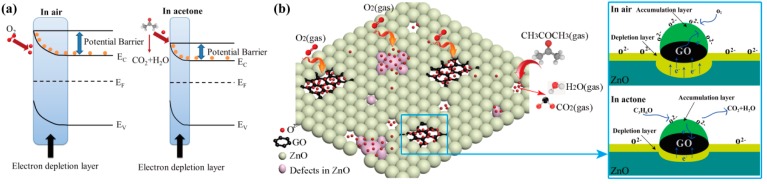
Schematic of (**a**) the depletion theory and (**b**) the sensing mechanism.

**Scheme 2:**
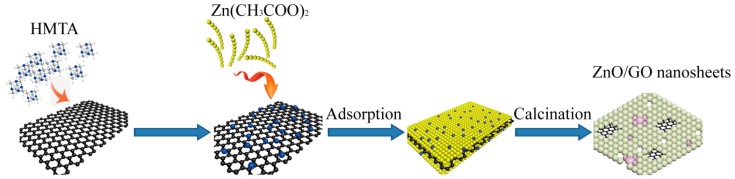
Schematic illustration for fabrication of ZnO/GO NSs-T using GO templated one step solution process. Step 1: preparation of GO by ultrasonic stripping of graphite oxide and the adsorption of HMTA on GO surface; Step 2: the adsorption of Zn(CH_3_COO)_2_ on GO surface; Step 3: Remove excess Zn(CH_3_COO)_2_ by washing; Step 4: the ZnO/GO NSs-T were prepared by calcination of precursor.

In this work, the ZnO/GO nanosheets were designed and used for acetone vapor detection. The high response, good selectivity, and quick response/recovery speeds were realized due to the ultra-thin nanosheets structure and the synergistic effects of ZnO and GO. The following reasons contribute to the unique sensing properties: (i) Large specific surface area of mesoporous nanosheets provided more active sites for the gas-surface. The large surface area of ZnO/GO NSs-525 means that there are large spaces for gas adsorption and desorption, and provide more active sites for surface contact reactions during the gas-sensing process [41]. The relationship between the surface to volume ratio with the gas sensitivity is described as Equation (4) [42]:

(4) $$S_{\phi }=\frac{\Gamma_{t}k_{Eth}\sigma_{0}C_{g}\phi }{n_{0}}\frac{V_{m}}{V_{s}}-1$$

where $\Gamma_{t}$ is a time constant, $C_{g}$ is the gas concentration, and $n_{0}$ is the electron concentration of the sensor at an operating temperature in the air atmosphere, $k_{Eth}$ is the reaction rate constant, $\sigma_{0}$ is a number of oxygen ion per unit area, ϕ is a ratio of surface area per volume of material (*V_m_*) and *V_s_* is the system volume. Hence, S_φ_ is linear with ($V_{m}/V_{s}$) $k_{Eth}$ and having a slope of $\Gamma_{t}\sigma_{0}C_{g}\phi /n_{0}$. Thus, for gas detection, the nanostructures register superior sensitivity with greater diffusivity paths for target gases and improved effective contact surface-to-volume ratio. (ii) The in situ modification of GO in ZnO/GO nanosheets after calcining can increase the adsorption of oxygen and increase the thickness of depletion layer. As shown in Scheme 1, the addition of GO will require the trapping of more electrons and reduce the thickness of the electron depletion layer. In addition, the depletion region around the GO might improve the modulation of nano-Schottky barriers during the oxidation of target gas, which also changes the carrier concentration and improves the sensing performances [43,44]. That is why the sensing properties are different between the ZnO/GO NSs-T samples with different GO amounts. Scheme 1 shows the depletion theory and the adsorption and reaction of surface oxygen species (O^2−^) with the target gas. As an n-type semiconductor, the oxygen species adsorbed on the surface led to the generation of an electron depletion layer on ZnO/GO NSs-525 and increased the surface potential barrier. By developing the electron-depleted surface layer, electrons were taken by the adsorbed oxygen species from the conduction band. Between ZnO and GO, the reaction of electron-transfer will be greatly promoted at the interface, because the accumulation layer on the GO side is acknowledged to attract large amounts of adsorbed oxygen. Once reducing gas such as acetone was contacted with the surface of sensing materials, they reacted with the adsorbed oxygen and released the trapped electrons back to the conduction band of ZnO, which in turn reduced the width of the electron-depleted layer and decreased the resistivity of ZnO [45]. The process is described in Scheme 2b and the reaction can be expressed as following [46]:

CH_3_COCH_3_ (gas) + 8O^2−^ (chemisorbed) → 3CO_2_ + 3H_2_O + 16e^−^ (5)

The selectivity of sensors was explained by the electron liberation theory, and the methanol and formaldehyde gases were taken as examples [46]:

CH_3_OH + 3O^2−^ → CO_2_ + 2H_2_O + 6e^−^ (6)

HCHO + 2O^2−^ → CO_2_ + H_2_O + 4e^−^ (7)

As a result, acetone can release much more electrons than those two in the same concentration from above formulas. That is why the ZnO/GO NSs-525 have better selectivity properties for acetone than other interference gases.

## 3. Materials and Methods

### 3.1. Materials

All of the chemicals were purchased from Aladdin Reagent Co. Ltd (Shanghai, China) without further purification before used. Graphite oxide was prepared according to the reported Hummers’ method [17].

### 3.2. GO Templated Synthesis of ZnO Nanosheets

Graphene-like porous ZnO/graphene oxide nanosheets were synthesized by the wet-chemical method (Scheme 2). In a representative process, 100 mg graphite oxide was dispersed into 200 mL absolute ethanol with the aid of ultrasonic to form a GO solution. Then the HMTA was added into. After ultrasonic treatment for 30 min, the zinc acetate with the molar ratio of (Zn:HMTA = 1:0.4) was added. The mixed solution was stirred overnight at 45 °C and then centrifuged it to get the product, washing several times with hot ethanol to remove excess Zn precursors and HMTA. The product was dried overnight. Subsequently, the powder was calcinated in a muffle furnace at different temperatures (475 °C, 500 °C, 525 °C, 550 °C, and 575 °C) with a heating rate of 1 °C/min and then kept the temperatures for 2 h. These samples were identified as ZnO/GO NSs-T (T = 475, 500, 525, 550, and 575).

### 3.3. Characterization

X-ray diffraction (XRD) patterns were measured by a Rigaku D/Max 2550 X-ray diffractometer with Cu-Kα radiation (λ = 0.154 nm, from 10–80°, 7°/min). Field emission scanning electron microscope (FE-SEM) (FEI, Quanta FEG 450, Hillsboro, OR, USA) was carried out to collect the morphology of the samples. Transmission electron microscopy (TEM) and selected area electron diffraction (SAED) images were performed by a Tecnai G220S-Twin transmission electron microscope (FEI, Hillsboro, OR, USA). Raman spectra were tested with an excitation wavelength of 532 nm through Raman Microscopy (Horiba, LabRAM HR Evolution, Paris, France). X-ray photoelectron spectroscopy (XPS,) studies were also analyzed through Thermo Scientific (Thermo Fisher Scientific, Waltham, MA, USA), ESCALAB 250. Brunauer-Emmett-Teller (BET) were measured by an ASAP-2020 gas adsorption analyzer with N_2_ as the absorbate and operation temperature at −196 °C. Thermogravimetric analysis (TGA) was determined by a thermogravimetric analyzer (TGA, PerkinElmer, Pyris1 Waltham, MA, USA) in the air (ramp = 10 °C/min).

### 3.4. Manufacture and Measurement of Sensors

Sensors were manufactured as reference [47]. Sensitive materials were mixed with ethanol and ground for 5 min to form a paste. Then, the paste was coated on the surface of a ceramic tube, which has a pair of Au electrodes with two Pt wires connected. After drying at 80 °C for 10 min, a thin sensing film was formed. To control the operating temperature, a Ni-Cr wire was carried for heating to insert it into the tube. Before the end, four Pt wires and the two ending of heating wire were welded onto a pedestal with six probes to get the gas sensor. Lastly, the sensor was matured by heating at 400 °C through the heating wire for 24 h. A CGS-8 series Intelligent Test Meter (Beijing, China) was carried out to test the sensing properties. The details of the measurement can be found in the reference [48].

## 4. Conclusions

In conclusion, ZnO/GO NSs-525 was prepared via a wet-chemical method successfully with graphene oxide as the template and sensitizer. HMTA was added into the solution to assist the formation of nanosheets in the product. Moreover, the amount of GO in ZnO/GO NSs-525 was controlled by changing the calcining temperatures. An extremely selective acetone vapor sensor based on the ZnO/GO nanosheets were reached by choosing 525 °C as the calcining temperature. Compared with the reported typical ZnO-based sensors, the ZnO/GO NSs-525 sensor exhibited a larger response value to acetone, with better selectivity and fast response/recovery speeds. The response value was 94 to 100 ppm acetone vapor. The synergistic effects of ZnO and GO nanosheets, including a unique 2-D structure, large specific surface area, suitable particle size, and the improved thickness of the depletion layer, contributed to the excellent sensing properties.

## Figures and Tables

**Table 1:** The sensing properties comparison of graphene-like porous ZnO/graphene oxide nanosheets with the reported acetone vapor sensors.

Materials	Concentration (ppm)	Response (Ra/Rg)	Recovery Time (s)	Refs
ZnO nanotube	100	3.5	10	[34]
Mesoporous ZnO	100	33	3	[35]
3D ZnO microsphere	100	22	17	[36]
ZnO/ZnCo_2_O_4_	100	7.5	36	[37]
Pt-ZnO-In_2_O_3_ nanofibers	100	57.1	44	[38]
Ce-ZnO nanoparticles	100	20	9	[39]
NiO/ZnO microflowers	100	23.5	41	[40]
ZnO/GO nanocomposites	100	35.8	7	[17]
ZnO/GO NSs	100	94	4	This work
